# Outcome-based health equity across different social health insurance schemes for the elderly in China

**DOI:** 10.1186/s12913-016-1261-5

**Published:** 2016-01-14

**Authors:** Xiaoting Liu, Hung Wong, Kai Liu

**Affiliations:** 1 Department of Social Security & Risk Management, School of Public Affairs, Zhejiang University, 866 Yuhangtang Road, Hangzhou, 310058 China; 2 Department of Social Work, The Chinese University of Hong Kong, Shatin, New Territories, Hong Kong, SAR China; 3 Department of Social Security, School of Labor and Human Resources, Renmin University of China, 59 Zhongguancun Street, Beijing, 100872 China

**Keywords:** Social health insurance, Health expenditure, Health outcome, Health equity, Elderly, China

## Abstract

**Background:**

Against the achievement of nearly universal coverage for social health insurance for the elderly in China, a problem of inequity among different insurance schemes on health outcomes is still a big challenge for the health care system. Whether various health insurance schemes have divergent effects on health outcome is still a puzzle. Empirical evidence will be investigated in this study.

**Methods:**

This study employs a nationally representative survey database, the National Survey of the Aged Population in Urban/Rural China, to compare the changes of health outcomes among the elderly before and after the reform. A one-way ANOVA is utilized to detect disparities in health care expenditures and health status among different health insurance schemes. Multiple Linear Regression is applied later to examine the further effects of different insurance plans on health outcomes while controlling for other social determinants.

**Results:**

The one-way ANOVA result illustrates that although the gaps in insurance reimbursements between the Urban Employee Basic Medical Insurance (UEBMI) and the other schemes, the New Rural Cooperative Medical Scheme (NCMS) and Urban Residents Basic Medical Insurance (URBMI) decreased, out-of-pocket spending accounts for a larger proportion of total health care expenditures, and the disparities among different insurances enlarged. Results of the Multiple Linear Regression suggest that UEBMI participants have better self-reported health status, physical functions and psychological wellbeing than URBMI and NCMS participants, and those uninsured. URBMI participants report better self-reported health than NCMS ones and uninsured people, while having worse psychological wellbeing compared with their NCMS counterparts.

**Conclusions:**

This research contributes to a transformation in health insurance studies from an emphasis on the opportunity-oriented health equity measured by coverage and healthcare accessibility to concern with outcome-based equity composed of health expenditure and health status. The results indicate that fragmented health insurance schemes generate inequitable health care utilization and health outcomes for the elderly. This study re-emphasizes the importance of reforming health insurance systems based on their health outcome rather than entitlement, which will particularly benefit the most vulnerable older groups.

## Background

After its reform and opening up, in the 1990s China encountered challenges to its market-oriented health care system reforms. In response to the increasing disparities in the health care system, since the beginning of the 21st century, the health insurance reform has begun to emphasize the concepts of health equity and social justice. The Chinese government initiated an ambitious reform plan in 2009, with the target of providing universal coverage and equitable access to health care by 2020. China’s huge and complex health care reform has gained early praise, especially for its demand-oriented social health insurance reform [1]. The social health insurance coverage increased from 29.7 % in 2003, to 87.9 % in 2008, and 95.7 % in 2011 [2]. It is remarkable to accomplish nearly universal coverage of social health insurance in such a short time. Additionally, the benefits offered by social health insurance are gradually rising in scope and in depth as well, which may contribute to mitigating the proportion of out-of-pocket payments made for, and the difficulties in accessing, health care. However, inequitable development between different insurance schemes is still a real threat to the objective of providing health for all and to the elimination of financial barriers to health care services.

As a result of these efforts, three major schemes have been launched to reformulate China’s social health insurance system, including the Urban Employee Basic Medical Insurance (UEBMI), the New Rural Cooperative Medical Scheme (NCMS), and the Urban Residents Basic Medical Insurance (URBMI), to target the cover of various social groups. Concretely speaking, the UEBMI was implemented in 1998 and aims at covering outpatients and inpatients services for urban employees. The premium is shared between employers and employees, while employers contribute about 6 % of gross payroll and employees paying for 2 % of their salary for each month to UEBMI. Its participants have better benefits packages than those in the other two systems. The retired employees aged 60 and over who already paid for minimum required years (20–30 years) are still covered by this scheme but stop to contribute premium to the fund pool while being supported by in-service employees. Statistically, 210.41 million employees and 72.55 million retirees are enrolled in the UEBMI by the end of 2014 [3]. Additionally, a traditional Socialized Medicine System providing free medical treatment to civil servants has been gradually reformed and merged into the UEBMI scheme. Therefore, we combine it with the UEBMI in this article. Unlike the relatively generous UEBMI, the first wave of the NCMS (for rural residents) and the URBMI (for the urban non-employed, such as students, children, the elderly and so on), initiated in 2003 and 2007 respectively, reimbursed only inpatient expenditures. At present, the coverage of the latter two schemes has gradually been expanded to outpatient costs. The elders living in rural areas and dependent on agriculture as the major livelihood are eligible for NCMS, and contribute fixed annual premium to the fund. Local governments have to annually subsidize participants with no less than their individual payments, and central government also providing subsidies to insured population in central and western regions. By the end of 2014, 736 million rural residents had joined the NCMS, accounting for a coverage rate of 98.9 % [4]. The elders who had no regular employment during their working life in urban areas are enrolled in URBMI, contributing a fixed annual premium and receiving governmental subsidies as well. The coverage of URBMI had reached 314.5 million at the end of 2014 [5]. In China, the participation of which insurance plan is not the result of self-selection. It is the determinants of the policies and exogenous variables, only related to domicile (rural or urban) and employment status, regardless of people’s socio-economic and other characteristics.

There are two concepts of health equity, “outcome-based health equality” (making an effort to pay more attention to the wellbeing of vulnerable elders) and “opportunity-based equality” (the equal opportunity for entitlement, which has been accomplished by universal coverage). Theoretically speaking, health equity should be defined as “outcome-based health equality”. In China’s previous health care reform, policy makers paid much more attention to opportunity equity, and expanded insurance coverage, in response to the problem of affordability. However, “outcome-based equity” was ignored in the policy formulation process, which damaged the health rights of vulnerable older adults. In addition, universal entitlement to health insurance does not guarantee equality in health care utilization and health outcome for each social group with similar demands for health care services, the equitable outcome of health care and health status is the ultimate objective of health insurance reform. On the one hand, although obtaining health insurance has some positive impacts on health care utilization [6–8], especially for old people [9, 10], these impacts are still limited in China [11]. One major concern is whether or not these insurance schemes are able to reach the elderly, who have a vulnerable socio-economic status, and improve the quality of their health care services [12, 13]. On the other hand, it is still a puzzle in the literature whether or not health insurance could improve health outcomes directly [14, 15]. All in all, the relationship between health insurance, health care expenditure and health outcome is still a question deserving further exploration, particularly in Chinese society. It is of interest whether or not diversified health insurance schemes have distinct effects on health equity. However establishing a causal link is complex because the measurement of health status is an imperfect process, and health insurance is only one factor that contributes to health equity [16]. Despite the difficulties, to make up the research gap, this article is aimed at exploring the association between different health insurance types and health outcome among the elderly. The empirical research findings derived from this study are expected to offer insight into the corresponding policy making process. And priority should be given to it to improve the comprehensive well-being of older adults in response to the trend of population ageing.

Most previous studies lack enough supportive data to clarify the diversified relations between health care and health status across different types of health insurance, and mainly focused on the correlation between socioeconomic status and health outcome. Actually, as mentioned above, social health insurance participation does not allow for self-selection and as a result of system requirements. To be specific, this research will unveil the disparities in health care expenditure and health status for the older adults in each insurance scheme by using ANOVA. After that, it will identify the effects of various insurance groups on the outcome of health status after controlling for socio-economic status (SES), social support, Hukou and chronic diseases through regression. Additionally, policy implications for the way forward will be discussed.

## Methods

### Data and sampling

This study drew upon data from the *National Survey of the Aged Population in Urban/Rural China* collected by the *China National Committee on Ageing* in 2006 and 2010. The sampling method *Probability Proportional to Size* (PPS) was adopted to select representative samples of those aged 60 or older, living in households in 2000 communities, in 160 counties/districts out of 20 provinces (Beijing, Hebei, Shanxi, Liaoning, Heilongjiang, Shanghai, Jiangsu, Zhejiang, Anhui, Fujian, Shandong, Henan, Hubei, Hunan, Guangdong, Guangxi, Sichuan, Yunnan, Shanxi, Xinjiang). One thousand samples were selected from each province in order to guarantee an adequate sample size. This data had been weighted according to its distribution in the *China Population Census Data*, which is available and can be generalized to the nationwide aged population. Generally, the survey done in 2006 consisted of 19947 valid samples while the later round survey conducted in 2010 was composed of 19986 older adults. Because these two surveys were just cohort rather than tracing studies, this article used the data collected both in the year of 2006 and 2010 for the ANOVA in order to compare group means before and after the new round of health care reform launched in 2009, and used the data collected in 2010 only for the multiple linear regression analysis to calculate associations between variables rather than causal relations. The data was used with permission from the *China National Committee on Ageing*.

### Measurements

#### Dependent variables

This paper used health care expenditure and health status to conceptualize health outcome. *Health care expenditure* contains three kinds of medical expenses for outpatient and inpatient services provided to aged people, including out-of-pocket spending, insurance reimbursement, and the total amount of the medical cost, to reflect the actual amount of utilization of health care. *Health status* was operationalized using three dimensions. The first one was self-reported health status measured by a five-point Likert-Scale, used to represent general health outcome, which predicted future mortality well [17]. Secondly, the Instrumental Activities of Daily Living Scale (IADLs) and the Activities of Daily Living Scale (ADLs) were used jointly to measure physical functions, aggregated from asking about sixteen items: eating, dressing, toilet hygiene, getting in and out of bed, cleaning, shopping, meal preparation, housework (washing), using a telephone, financial management, bathing and showering, functional mobility in the room, going up and down stairs, taking a bus, walking 3–4 li (500 m per li), and lifting 10 kg weights. A 3-point response scale was used to measure different levels of capacity for each item: unchallenged, somewhat challenged, and incapable. The Cronbach’s Alpha for this scale was 0.927 and 0.942 for the samples in 2006 and in 2010 respectively. Thirdly, psychological wellbeing should not be ignored as a dimension of health outcome. It was measured by a Worry Degree Scale, comprised of nine items: have nothing to live for; have no money to treat an illness; nobody could provide care when get sick; social insecurity; children do not pay filial respect to parents; insufficient pension; children are unemployed; unsafe transportation; and could not recover from disease. The Cronbach’s Alpha for this scale was 0.889 and 0.906 for the 2006 and the 2010 samples respectively. The Pearson correlation coefficient between physical functions and self-reported health status is 0.506, that of psychological wellbeing and self-reported health status is 0.214, and physical functions have a low correlation coefficient of 0.102 with psychological wellbeing.

#### Independent variables

The core independent variable was *social health insurance,* including the above mentioned three types of insurance schemes, the UEBMI, the URBMI and the NCMS, as well as the uninsured group. Commercial insurance was excluded from this study due to the small fraction of participants compared with the nearly universal coverage of the social health insurance schemes. Some predictive variables were enrolled in the regression model for control. Firstly, income and education were used as indicators of *socio-economic status (SES)* [18]. Occupation was excluded from the model as most of the aged population had dropped out of the labour market based on a nationally compulsory retirement age of 60 years old for men, 50 years old for female workers and 55 years old for female cadres. *Individual income* was calculated from multiple sources: income from work (e.g. salary, agricultural income), social security (e.g. pension, annuity), governmental allowances (e.g. social assistance, old age allowance), financial income (e.g. rental, stock, interest), and transferred income (e.g. money from children or other relatives).

Secondly, *social support* was supposed to be a significant predictor of the health outcome of older adults both in Chinese and Western contexts [19, 20]. More specifically, receiving social support has positive effects not only on physical health [21] and psychological health [22, 23], but on the life satisfaction and the quality of life for the elderly [24, 25]. In this article, social support from networks was conceptualized by using three components: connecting support, emotional support and substantial support. The connecting support was measured by the number of relatives and friends that you connected with at least once a month, with the emotional support being measured by the number of relatives and friends who could have a heart-to-heart talk with you, while substantial support was measured by the number of relatives and friends who could help when you needed it. The Cronbach’s Alpha for this social support scale was 0.872 for the samples taken in 2006 and 0.816 for those in 2010.

Thirdly, it was necessary to take into account the *hukou* (household registration) when we focused on the health equity issue in China. As 80 % of the government health expenditure was concentrated in urban areas, actually, it remained controversial whether or not inequalities in health between urban and rural regions had been narrowed [26, 27]. Similarly, the hukou was consistently associated with inequitable accessibility and expenditure of health care due to inequitable distribution of health resources [27, 28]. However, the hukou was removed from the regression model because of a problem with multi-collinearity with the URBMI and the NCMS.

Furthermore, *chronic diseases* increased rapidly along with the ageing population, resulting in increasing disabilities for the elderly. The longitudinal samples used in this study informed that the morbidity rate for chronic disease had increased from 75.0 % (urban: 81.2 %; rural: 68.8 %) in the year of 2006 to 77.4 % (urban: 82.8 %; rural: 72.1 %) in 2010, both in urban and rural areas. The average number of chronic diseases each older person suffered from had jumped from 1.96 to 2.85 during the 5 years between the surveys. Accordingly, this research included the number of chronic diseases in the model. In addition, another potentially confounding variable, the *severity of illness,* was also controlled for in our regression model.

### Data analysis

Ahead of inferential statistics, the characteristics of the samples were briefly described, followed by a one-way ANOVA comparing the group means of health outcome and health care expenditures between insurance schemes for the year of 2006 and 2010. After that, a Multiple Linear Regression was applied as well to identify the effects of different types of health insurance on health outcomes after the new round of health care reforms initiated in 2009, if we controlled for above measured confounding variables. We looked at the distribution of dependent variables of health outcome, which is the basic principle of using linear regression. After checking the distribution of health outcomes, self-reported health status (skewness = −.097; kurtosis = .282) and psychological well-being (skewness = −.077; kurtosis = .282) obey the rule of normal distribution well. After deleting the outliers, the value of skewness for physical functions is around −1, which is acceptable. Only the data collected in 2010 was used in the models as it was not a longitudinal survey testing the characteristics of the same person in both rounds.

## Results

### Sample characteristics

According to Table 1 giving some socio-demographic variables, the average age of the persons sampled increased from 71.18 in 2006 to 72.25 in 2010, owing to the trend of population ageing. In general, the education level had improved as the illiteracy rate decreased by 19.13 % while the number of elders with diplomas from middle school and above rose quickly. Furthermore, individual income nearly doubled due to a dramatic increase in social security income from 2006 to 2010. Specifically, as a nationwide New Rural Social Pension Policy was initiated in 2009, the proportion of rural samples not having any income decreased from 81.78 to 46.45 % during 2006 to 2010, whose means of individual income raised from CNY 26.82 to CNY152.40, totally increased 4.68 times for rural old residents. In the urban areas, the rapid growth of pension benefits was another contribution to the obviously increased individual income for the elderly, the means of which for urban samples increased from CNY 1078.72 to 1612.15, totally 49.45 % from 2006 to 2010. Furthermore, as the process of urbanization accelerated, the proportion of urban samples increased to 50.3 % in 2010, whereas the percentage had only been 49.8 % for the samples taken in 2006. Moreover, the average number of family members, relatives and friends who provide social supports separately increased 12.05 for 2006 and 19.80 for 2010.

**Table 1:** Sample characteristics

	2006	2010
Characteristics	*n* = 19947	*n* = 19986
Age: mean (SD^a^)	71.18 (7.00)	72.25 (7.45)
Range (years)	60–109	60–103
Sex: n (%)	19947 (100)	19986 (100)
Men	10462 (52.4)	10338 (51.7)
Women	9485 (47.6)	9648 (48.3)
Education: n (%)	19929 (100)	19957 (100)
Illiteracy	7146 (35.9)	5779 (29.0)
Primary school	7255 (36.4)	7772 (38.9)
Junior middle school	2918 (14.6)	3635 (18.2)
Senior middle school/Technical secondary school	1664 (8.3)	1780 (8.9)
University and above	946 (4.7)	991 (5.0)
Individual income^b^: mean (SD)	483.35 (963.45)	886.61 (1689.56)
Hukou: n (%)	19914 (100)	19973 (100)
Urban	9920 (49.8)	10054 (50.3)
Rural	9994 (50.2)	9919 (49.7)
Numbers of chronic disease: mean (SD)	1.96 (2.00)	2.85 (1.92)
Range	1–16	1–25
Social support: mean (SD)	12.05 (7.13)	19.80 (18.81)
Health insurance: n (%)	18662 (100)	19532 (100)
UEBMI	6328 (33.9)	7423 (38.0)
URBMI^c^	——	1853 (9.5)
NCMS	4698 (25.2)	9675 (49.5)
Uninsured	7636 (40.9)	581 (3.0)

^a^ *SD* standard deviation. ^b^As identified in the measurement, individual income includes income from work, social security, governmental allowances, financial income, and transferred income. ^c^URBMI was initiated in 2007, that is why there is no data for 2006

### The one-way ANOVA: health inequity between insurance schemes

#### Health insurance and health expenditures

Over the last decade, social health insurance schemes have been continuously expanded to mitigate the rise in out-of-pocket payments and to deal with the lack of equity in the financing of, and access to, health care [29]. The coverage rate of the NCMS System for old rural residents climbed from 44.7 % in 2005 to 98.3 % in 2010. Health insurance coverage for their counterparts, old urban residents, increased from 74.1 % in 2005 to 95.3 % in 2010 [30]. At the same time, achieving health equity among the sub-groups of various insurance schemes is still a big challenge despite the nearly universal coverage of health insurance, and this has not been studied extensively. The number of rural elderly enrolled in the NCMS rose dramatically from 25.2 to 49.5 %, while the uninsured elderly decreased quickly to only 3 %, as indicated in Table 1. The coverage of UEBMI participants also rose from 33.9 to 38.0 % within the given time frame.

A one-way ANOVA was conducted in order to determine whether or not there was a significant variance in the means of health care expenditures across diversified insurance groups in both the 2006 and the 2010 samples (Table 2). For the samples taken in 2006, we were able to conclude that the three groups (UEBMI, NCMS and uninsured persons) were different with respect to the means of their total health expenditures (F [3, 19943] = 364.21, *p* < 0.001), insurance expenditures (F [3, 19943] = 537.75, *p* < 0.001), and Out-of-pocket costs (F [3, 19943] = 54.06, *p* < 0.001). The results for the samples from 2010 were similar: the four groups (UEBMI, URBMI, NCMS and uninsured old people) had different means of total health expenditures (F [4, 14050] = 207.70, *p* < 0.001), insurance expenditures (F [4, 13859] = 355.38, *p* < 0.001), and out-of-pocket expenses (F [4, 13859] = 76.29, *p* < 0.001). It should be noted that URBMI was initiated in 2007, and therefore produced no relevant data in 2006. In addition, UEBMI covered both inpatient and outpatient benefits initially, whereas the outpatient reimbursements for NCMS and URBMI beginning at 2009, the cost of which being missed in the insurance expenditures of 2006.

**Table 2:** The disparities in health care expenditure by Insurance Schemes

	Total health care expenditure (CNY)		Insurance expenditure^f^ (CNY)		Out-of-pocket cost (CNY)	
	2006	2010	2006	2010	2006	2010
UEBMI^a^	4217.77	8089.14	2627.8 (62.30 %)^d^	5059.74 (62.55 %)	1416.77 (33.59 %)	2638.17 (32.61 %)
URBMI^b^	——	4013.44*	——	1255.14* (31.27 %)	——	2458.50* (61.26 %)
NCMS	1005.71*	1899.80*	111.79* (11.12 %)	526.26* (27.70 %)	855.36* (85.05 %)	1255.95* (66.11 %)
Uninsured	1099.14*	3362.22*	0*	0*	992.05* (90.26 %)^e^	2660.23* (79.12 %)
Total^c^	2100.68***	4460.44***	912.05*** (43.42 %)	2295.79*** (51.47 %)	1089.3*** (51.85 %)	1927.95*** (43.22 %)
Difference						
UEBMI	Reference	Reference	Reference	Reference	Reference	Reference
URBMI	——	4075.70	——	3804.6	——	179.67
NCMS	3212.06	6189.34	2516.01	4533.48	561.41	1382.22
Uninsured	3118.63	3628.70	2627.80	5059.74	424.72	−22.06
Relative ratio						
UEBMI	Reference	Reference	Reference	Reference	Reference	Reference
URBMI	——	2.02	——	4.03	——	1.07
NCMS	4.19	4.26	23.51	9.61	1.66	2.10
Uninsured	3.84	2.41	——	——	1.43	0.99

^a^In the Post Hot Tests, this table only presented the significant level of difference in each two groups’ means if taking UEBMI as a reference; * the mean difference is significant at the 0.05 level

^b^As URBMI was launched in 2007, there is no data for this insurance plan in 2006

^c^***p < 0.001, at least one group had a significantly different mean of total health expenditures, insurance expenditures and out-of-pocket expenses

^d^The percentage in brackets demonstrated the proportion of insurance expenditures/ out-of-pocket cost accounted for total healthcare expenditures in each insurance scheme

^e^The reason why out-of-pocket did not account for 100 % of total healthcare expenditures for uninsured group was that partially covered by Medical Assistance System

^f^The insurance expenditures of UEBMI included both outpatient and inpatient costs in 2006 and 2010; which of URBMI and NCMS contained only inpatient reimbursements in 2006 and both inpatient and outpatient benefits in 2010 as the policy changed in 2009

A post hoc test was then employed to identify where significant differences existed. Two summary measures of health inequality were also designed to determine the absolute and relative magnitude of these differences, with the larger number representing more inequities. With regard to the total health care expenditures, for instance, results of the analysis indicated that the mean costs of UEBMI participants markedly exceeded those of the NCMS, the URBMI and uninsured groups. The means of the total health care expenditures showed an obvious growth from 2006 to 2010, with growth rates of 91.79 and 88.90 % for UEBMI and NCMS participants respectively. Both the absolute difference and the relative ratio between the UEBMI and the NCMS enlarged, while the relative ratio between the UEBMI and the uninsured reduced from 2006 to 2010. Similarly, compared with the insurance reimbursements in 2006, those of the NCMS increased 3.71 times from 2006 to 2010; while the UEBMI also raised its benefits 1.93 times the baseline during those 5 years. The UEBMI reimbursed the largest proportion of total medical costs as its benefits packages were significantly better than those of the URBMI and the NCMS. Although the absolute difference between the UEBMI and the NCMS was inflated from 2006 to 2010, the relative ratio between them decreased. In the meantime, by comparing the group means, out-of-pocket expenditures were more than insurance reimbursements for URBMI and NCMS both in 2006 and 2010, indicating a severe challenge to cost control for our health care service system. Some studies also proposed a warning that health insurance might increase, rather than dispersing, financial risk by arousing demand for care [31]. In addition, although insurance coverage was nearly universal, the out-of-pocket spending for the 3 % uninsured older adults increased 168.15 % during the two-rounds of investigation.

### Health insurance and health outcome

We checked the variation in health outcome between and within health insurance groups, using a one-way ANOVA as well, and found that at least one group was different in its means of health outcome with that in other groups. According to Table 3, the average self-reported health status, physical functions and psychological well-being of UEBMI respondents were statistically better than those of the URBMI, NCMS, and even uninsured groups in both of the two designated years (at the 5 % significance level). Fortunately, health equity was enhanced when we compared the group means between NCMS participants and the uninsured group. There were statistically significant disparities in all three indicators of health outcome in 2006 (at the 5 % significance level), but the differences in physical health and self-reported health status between various insurance schemes became statistically insignificant in 2010. Table 3 also illustrates that the means of the physical functions of all three insured groups had slightly declined while their psychological well-being had increased gently during the 5 years’ period. The relative ratio of the three health outcome indicators between the UEBMI and other groups changed little from 2006 to 2010, except for the ratio between the UEBMI and the NCMS which increased from 1.03 in 2006 to 1.14 in 2010.

**Table 3:** Comparison of group means of health outcome among different types of health insurance schemes

	Self-reported health status^a^		Physical functions^a^		Psychological well-being^a^	
	2006	2010	2006	2010	2006	2010
UEBMI^b^	3.1	3.1	39.42	38.58	26.21	29.88
URBMI	——	2.89*	——	36.61*	——	24.92*
NCMS	2.96*	2.89*	37.88*	37.13*	25.39	26.22*
Uninsured	2.88*	2.91*	37.12*	36.58*	21.82*	24.65*
Total^c^	2.98***	2.97***	38.09***	37.62***	25.17	27.47***
Relative ratio						
UEBMI	Reference	Reference	Reference	Reference	Reference	Reference
URBMI	——	1.07	——	1.05	——	1.20
NCMS	1.05	1.07	1.04	1.04	1.03	1.14
Uninsured	1.08	1.07	1.06	1.05	1.20	1.21

^a^Self-reported health status is measured by a five-point Likert-Scale, while the range of the physical functions scale and the psychological well-being scale are 0–48 and 0–27 respectively. Higher value means better health status in all of above three dimensions of health outcome

^b^In the Post Hot Tests, this table only presented the significant level of difference in each two groups’ means if taking UEBMI as a reference; * the mean difference is significant at the 0.05 level

^c^***p < 0.001, at least one group was different in its means of health outcome with that in other groups

### Multiple Linear Regression: health insurance as a predictor of health outcome

We obtained both consistent and inconsistent findings with previous evidence through the Multiple Linear Regression analysis. Three dimensions of health outcome, composed of self-reported health status, physical functions, and psychological well-being, constituted dependent variables and were predicted separately. The statistical results for the different dimensions of health outcome are shown in Tables 4, 5 and 6 respectively. Each table includes four models, each model setting out a different insurance status one by one to act as the reference group. Standardized coefficients are shown in the tables. We primarily discussed coefficients of the major independent variables that is the insurance status of the different participants, to focus on health equity. We reported the coefficients of the control variables subsequently.

**Table 4:** Regression results for the self-reported health status of the elderly in 2010

	Model 1	Model 2	Model 3	Model 4
	Beta (SE)	Beta (SE)	Beta (SE)	Beta (SE)
Age	−0.140 (0.001)***	−0.141 (0.001)***	−0.141 (0.001)***	−0.141 (0.001)***
Sex (male = 1)	0.016 (0.014)	0.016 (0.014)	0.016 (0.014)	0.016 (0.014)
Education	0.085 (0.008)***	0.081 (0.008)***	0.081 (0.008)***	0.081 (0.008)***
Individual income	0.047 (0.000)***	0.044 (0.000)***	0.045 (0.000)***	0.044 (0.000)***
Numbers of chronic disease	−0.236 (0.004)***	−0.236 (0.004)***	−0.236 (0.004)***	−0.236 (0.004)***
Social support	0.067 (0.000)***	0.067 (0.000)***	0.066 (0.000)***	0.066 (0.000)***
UEBMI	Reference	0.058 (0.024)***	0.111 (0.018)***	0.083 (0.036)***
URBMI	−0.026 (0.024)**	Reference	0.036 (0.023)***	0.020 (0.037)
NCMS	−0.106 (0.018)***	−0.054 (0.023)***	Reference	−0.029 (0.035)
Uninsured	−0.019 (0.042)*	−0.003 (0.044)	0.013 (0.041)	Reference
Adjust R Square	0.121	0.122	0.122	0.122
Observations	13709	13709	13709	13709

UEBMI is used as a reference in Model 1, URBMI as a reference in Model 2, NCMS as a reference in Model 3, and the uninsured group as a reference in Model 4

*SE* Standard error

***p < 0.001; **p < 0.01; *p < 0.05

**Table 5:** Regression results for physical functions of the elderly in 2010

	Model 1	Model 2	Model 3	Model 4
	Beta (SE)	Beta (SE)	Beta (SE)	Beta (SE)
Age	−0.404 (0.007)***	−0.404 (0.007)***	−0.404 (0.007)***	−0.404 (0.007)***
Sex (male = 1)	0.032 (0.100)***	0.031 (0.100)***	0.031 (0.100)***	0.031 (0.100)***
Education	0.087 (0.055)***	0.084 (0.055)***	0.084 (0.055)***	0.084 (0.055)***
Individual income	0.023 (0.000)*	0.020 (0.000)*	0.020 (0.000)*	0.020 (0.000)*
Numbers of chronic disease	−0.159 (0.025)***	−0.159 (0.025)***	−0.159 (0.025)***	−0.159 (0.025)***
Social support	0.047 (0.002)***	0.046 (0.002)***	0.046 (0.002)***	0.046 (0.002)***
UEBMI	Reference	0.076 (0.175)***	0.093 (0.133)***	0.092 (0.260)***
URBMI	−0.041 (0.173)***	Reference	0.011 (0.164)	0.010 (0.269)
NCMS	−0.088 (0.132)***	−0.017 (0.164)	Reference	−0.001 (0.252)
Uninsured	−0.028 (0.303)***	−0.006 (0.316)	−0.001 (0.296)	Reference
Adjust R Square	0.247	0.247	0.247	0.247
Observations	13529	13529	13529	13529

UEBMI is a reference in Model 1, URBMI is a reference in Model 2, NCMS is a reference in Model 3, and the uninsured group is a reference in Model 4

*SE* standard error

***p < 0.001; *p < 0.05

**Table 6:** Regression results for the psychological well-being of the elderly in 2010

	Model 1	Model 2	Model 3	Model 4
	Beta (SE)	Beta (SE)	Beta (SE)	Beta (SE)
Age	0.118 (0.012)***	0.117 (0.011)***	0.117 (0.011)***	0.116 (0.011)***
Sex (male = 1)	−0.042 (0.171)***	−0.042 (0.171)***	−0.042 (0.171)***	−0.042 (0.171)***
Education	0.098 (0.093)***	0.093 (0.094)***	0.092 (0.094)***	0.093 (0.094)***
Individual income	0.088 (0.000)***	0.084 (0.000)***	0.083 (0.000)***	0.084 (0.000)***
Numbers of chronic disease	−0.112 (0.043)***	−0.112 (0.043)***	−0.112 (0.043)***	−0.112 (0.043)***
Social support	0.073 (0.004)***	0.073 (0.004)***	0.073 (0.004)***	0.073 (0.004)***
UEBMI	Reference	0.184 (0.300)***	0.107 (0.226)***	0.195 (0.449)***
URBMI	−0.104 (0.297)***	Reference	−0.048 (0.282)***	0.004 (0.464)
NCMS	−0.094 (0.224)***	0.081 (0.283)***	Reference	0.092 (0.436)***
Uninsured	−0.063 (0.522)***	−0.010 (0.546)	−0.034 (0.511)***	Reference
Adjust R Square	0.08	0.081	0.081	0.081
Observations	13140	13140	13140	13140

UEBMI is a reference in Model 1, URBMI is a reference in Model 2, NCMS is a reference in Model 3, and the uninsured group is a reference in Model 4

*SE* standard error

***p < 0.001

Firstly, with regards to self-reported health status, the UEBMI participants had significantly higher self-reported health status than the URBMI and NCMS participants and the uninsured people, as shown in Model 1 of Table 4. Compared with their URBMI counterparts, NCMS participants had significantly 0.054 units of lower self-reported health status in Model 2 of Table 4. If taking NCMS as reference, UEBMI insured elders had a 0.111 units of higher self-reported health level, simultaneously, URBMI participants had 0.036 units of better health outcome. No significant difference was found between URBMI and NCMS participants and those uninsured in Model 4. Secondly, in terms of physical functions, UEBMI insured participants similarly presented significantly better physical functions compared to the other three groups in Model 1 of Table 5. If controlling for other variables, URBMI insured elders had 0.041 units of worse physical health than UEBMI participants, and NCMS having 0.088 units in the same circumstances. The differences between the other three groups were all statistically non-significant (Refer to Model 2–4 in Table 5). Thirdly, UEBMI participants also had significantly better psychological well-being than the other three groups in Model 1 of Table 6. However, differing from the results for self-reported health status, NCMS participants had 0.081 units of better psychological well-being than the URBMI insured groups and 0.092 units of that for uninsured people in Model 2, Model 3 and Model 4, and no significant difference was found between URBMI participants and uninsured people in Model 4 of Table 6. It should be interpreted cautiously as the psychological model had a lower explanatory power.

The results could be summed up using a simple ranking. The self-reported status of UEBMI participants ranked first among the four groups, that of URBMI ones second, and that of NCMS ones and uninsured people third with no significant difference between the two of them; the physical functions of UEBMI participants ranked first, and the other three groups, with no significant difference between them, ranked second; finally, the psychological well-being of UEBMI participants still ranked first, that of NCMS ones second, and that of URBMI scheme participants and uninsured older people third with no significant difference between the two of them. Consequently, UEBMI was the most important factor to predict better health for the elders in each model compared to other two social insurance plans for rural and urban residents, even though SES and other related variables were controlled. The probable reason was that UEBMI provided best benefits to the participants, reflected from the comparison in group means of insurance expenditures in the ANOVA part, which played a protective effect for health outcome. This mechanism would be discussed in the next part.

Beyond the effects of insurance types across all models, either age or sex demonstrated opposite relationships with physical and psychological health status. Tables 4 and 5 also separately showed a strong negative association between age and self-reported health status as well as physical functions. For example, in Model 1 of Table 4, the self-reported health status would decrease 0.14 units along with 1 year of age growth. On the contrary, in Model 1 of Table 6, as age increased 1 year, psychological well-being significantly improved 0.118 units accordingly. Simultaneously, males reported 0.032 units of better physical functions than female (Model 1 in Table 5), while reporting 0.042 units of worse psychological well-being than females did (Model 1 in Table 6). The possible reasons might be a greater tendency for men rather than women to be living independently during later life, or that most men, rather than women, were bread-winners, who had responsibility for taking care of family members.

Other variables indicated the same direction of association with physical and psychological health outcome in each model. SES (education and income) demonstrated a significantly positive effect on health outcome, consistent with previous literature. Furthermore, by comparing standardized coefficients, social support was another primarily successfully explanatory variable for health outcome. Taking physical functions model as an example, if social support increased one unit, the physical health for the elders would significantly improve 0.047 units (see Model 1 in Table 5). Simultaneously, the number of chronic diseases had a negative effect on self-reported health status, physical function, and psychological health. Finally, the factor of the hukou (household registration) was excluded from the models just because of a multicollinearity problem with the various insurance types, which would be discussed in the next section.

Ultimately, the three dimensions of health outcome suggested using some discrimination in interpreting the variation in health outcomes in the different models. Specifically, Model 1 in Table 4 shows that 12.1 % of the variation in self-reported health status could be interpreted by age, gender, SES (education and income), number of chronic diseases, social support, and social health insurance (URBMI, NCMS and uninsured). The models in Table 5 were able to explain the much higher variation (24.7 % in each model) in physical functions. However, only 8 % variation in psychological well-being was expected to be successfully interpreted by the psychological well-being models in Table 6. The possible explanation why psychological well-being model has a lower R Square might be that physical disease oriented social medical insurance system in China covers less mental health services and drugs. This institution might mainly impact on physical health rather than psychological health for the elderly in China. Likewise, there was a similar capacity for interpretation regarding the three batches of models using the survey data collected in 2006.

## Discussion

Evidence from previous studies pointed out that having insurance does increase health care utilization [10]. However, these studies did not take into account the disparities between various insurance schemes in the distinctive social context of China. This study sheds new light on disparities in health outcomes between participants enrolled in divergent health insurance plans. Older recipients of the UEBMI with higher SES (stable job as well as high education and income level) are eligible for more health care resources and correspondingly have better health outcomes than insured elderly people covered by the NCMS, the URBMI, and those in the uninsured group. Fortunately, during the process of health care reform, the proportion of out-of-pocket costs accounting for total healthcare expenditures had gradually decreased from 2006 to 2010, in the same time, the percentage of insurance expenditures increasing in each insurance scheme. In particular, the reimbursement rate of NCMS for the elders rose up from 11.12 to 27.70 %, according to Table 2, resulting in 19.94 % descended in out-of-pocket costs during the 5 years between the surveys. Meanwhile, the gap of reimbursement benefits between the UEBMI and the NCMS was reduced, although their absolute values were still rising. Absolutely, a remarkable achievement of improving health equity, especially the alleviation of disease burden for vulnerable elders, was gained from healthcare reform.

Nevertheless, another story revealed the underlying problems of health inequities between diversified health insurance schemes. Participants in the latter two insurance schemes have to pay more out-of-pocket expenses than the amount of money they get as reimbursements from insurance plans, according to the comparison of the group means by the ANOVA. Especially, the out-of-pocket expenses paid by the uninsured even exceeded that by the UEBMI participants in 2010, revealing a major financial hardship that the uninsured group suffered from. Much literature also identified individual financial hardship caused by the growth of out-of-pocket spending in China, especially in the case of catastrophic medical expenses leading to a high probability of impoverishment [13, 32]. By descriptive analysis, the means of out-of-pocket expenses paid by the uninsured, UEBMI and NCMS groups increased by 168.15, 86.21 and 46.83 % respectively. This result illustrates that more concern should be attached to out-of-pocket costs, increasing more substantially, at the same time that health insurance reimbursements increasing gradually. Also, the acquisition of insurance coverage may lead to the growth of out-of-pocket spending as well [31, 33], which should be given much more attention in future studies. Besides basic coverage, the benefits package of the NCMS and URBMI should be raised as the deductibles are high, ceilings are low, and co-payment rates for individuals are not low enough.

In the endeavour to achieve outcome-based health equity, one major concern is whether or not health insurance is able to improve health outcomes for the vulnerable elderly. The research findings derived from this study show distinctive associations between different types of health insurance and self-reported health status, physical functions and psychological well-being, despite it not being a causal relationship. Beyond the absolute predominance of the UEBMI across all models, the analysis demonstrates that URBMI attendants have a better self-reported health status while having worse psychological well-being than those attendants who are eligible for the NCMS. The disparities in self-reported health status between urban and rural residents have been verified in previous literature [26, 34]. However, the elderly enrolled in the URBMI suffer from a series of potential risks, such as insufficient money paid for their healthcare, no caregiver providing care for the aged, lack of adequate pension, and unsafe transportation. Against the background of the current social transition, it is of interest that there are more psychological risk factors for urban vulnerable old people (e.g., they are excluded from the better UEBMI system due to their not being retirees from state-owned companies or other stable job positions) than there are for NCMS rural senior residents. To some extent, the psychological well-being of the urban disadvantaged old population as reflected by the URBMI attendants should be of concern to policymakers. In this respect, we should not use universal coverage to cover up disparities among these different insurance schemes during the process of health insurance reform. Another outcome-based policy, targeted at equitable health outcome, should be announced to improve the health outcome of the NCMS, the URBMI participants, and even the elderly who have not any insurance coverage yet.

Meanwhile, as it is complicated for health outcome, we are aware of the limited effect of health insurance on health equity to a certain extent, even though adopting an experimental or quasi-experimental methods to explore their causal relations [14]. In addition, these existing inequalities of health outcome across different insurance schemes, although statistically significant, are of moderate or even small magnitude. The relative ratio between the UEBMI and other groups is minor. In that case it is necessary to introduce a perspective of life course to explain the production of health inequities over an individual’s lifetime. According to a widely used accumulation model, risk factors at different life stages accumulate over time, in the process of which formed personal health trajectories for disadvantaged groups, including the NCMS, the URBMI and uninsured groups [35, 36]. The current health inequities also manifest the inequalities of education and labor trajectories in earlier stages of life course, not necessarily attributable to the social health insurance scheme that covers them at their present age. It is well known that UEBMI insured group has the better education level and stable jobs, which were obtained in the stage of childhood, adolescence, and working periods.

Besides health insurance, other social determinants such as social support also have an impact on health outcome in the regression models. With regard to the social determinants, this study, consistent with previous literature, shows that the receipt of social support has a significant impact on equitable health outcome. It draws attention to the importance of protecting social networks for the elderly in future health insurance reforms, as our social health insurance schemes protect individuals only rather than supporting family members who are major sources of social support for the elderly.

In addition, although we removed the hukou from the regression model due to the problem of multicollinearity with different types of insurance plans, the NCMS could be regarded as a replacement for the hukou as 98.3 % of the rural residents are covered by this scheme in the descriptive analysis. Actually, the disparities in the group means of health outcomes and health expenditures between rural and urban residents continued to exist, according to a T-test, even after the health care reform launched in 2009. Though a large amount of money was invested in rural areas during the promotion of health equity, it is noted that there is little evidence of a remarkable improvement in health outcomes due to help from the healthcare reform.

## Policy implication

This study contributes to a concept shift away from opportunity-oriented to outcome-based health equity. A common feature of our health care reform is to use more and more public health expenditure as a solution to China’s health care problems, while spending much more money may not necessarily lead to a better outcome. The continuous expansion of health insurance will no doubt improve accessibility, but critical questions about the efficiency, quality of care and financial sustainability will remain unaddressed unless we look at it from a perspective based on outcome-based health equity.

How could the outcome-based strategy be formalized? Firstly, outcome-based health equity is constructed on the basis of equitable insurance benefits for all social groups, especially increasing reimbursements for URBMI and NCMS, and shrinking the gaps with UEBMI. Secondly, in the future health insurance reform, the role of insurance agency should be changed from payer for health care costs to the health manager. Insurance funds will be paid according to performance of improving health outcome rather than just amount of medical expenditures, which will be helpful for cost control and health equity. Thirdly, health outcome could be evaluated not only with physical health status but also self-reported health status and psychological well-being. Both objective and subject health outcome measurement will be equally treated to improve the comprehensive health status for the elders.

Based on outcome-oriented health equity, a comprehensive health care model, integrating long-term care, medical care, primary care, and even social care, should be developed, with primary care in the communities being especially recommended [37]. The traditional medical treatment model does not adapt to population ageing and challenges caused by chronic diseases. An integrated model combining medicine and elderly care will be further studied in the near future.

The findings from this article should be interpreted cautiously as there are several limitations that should be acknowledged. Firstly, although two rounds of survey data are employed, they are only cohort rather than panel data. This causes difficulties with inferring a causal association between health insurance and health outcome. Secondly, except for its direct effect on health care expenditures and health outcome, health insurance might have a mediating or moderating effect on associations between other predictive variables and health outcome, which is not considered in this study. Additionally, neither health insurance nor health outcome can be measured completely without measurement error due to the complexity of the concepts. Last but not least, as local governments take major responsibility for the financing and management of health insurance schemes, variation between regions should be taken into account in future studies.

## Conclusions

All in all, the research findings of this paper contribute to our understanding of the inequities in health outcomes of the elderly between different social health insurance schemes in China, and critically promote a conceptual shift from opportunity-oriented to outcome-based equity in the process of health care reform. This advocates efforts to improve quality and outcome rather than merely accessibility in future reforms. However, there is still a long journey ahead of it. Fortunately, over the past decade, health care reform has ensured affordable access to health care services with the achievement of nearly universal health insurance coverage. This research, drawing upon nationally representative data, partially reveals the remaining gaps in health care expenditures and health outcomes among different insurance schemes designed for social groups with diversified identities, especially after China’s new round of health care reforms carried out in 2009. After the expansion of insurance coverage, it is an even greater challenge to bridge gradually the gap in benefits between the three major basic insurance schemes and reduce the out-of-pocket expenses of vulnerable older adults.

## Abbreviations

NCMS: New Rural Cooperative Medical Scheme
SES: socio-economic status
UEBMI: Urban Employee Basic Medical Insurance
URBMI: Urban Resident Basic Medical Insurance
